# Negative regulators antagonizing antitumor innate immune pathways in lung cancer immune evasion

**DOI:** 10.3389/fimmu.2026.1842558

**Published:** 2026-05-11

**Authors:** Fan Yang, Rui Liu, Li Liu, Aijie Zhang, Bo Li

**Affiliations:** 1 Department of Oncology, Suining Central Hospital, Suining, Sichuan, China; 2 Department of Breast and Thyroid Surgery, Suining Central Hospital, Suining, Sichuan, China; 3 Basic Laboratory Suining Central Hospital, Suining, Sichuan, China; 4 Department of Respiratory and Critical Care Medicine, Suining Central Hospital, Suining, Sichuan, China

**Keywords:** cGAS–STING, immune evasion, innate immunity, lung cancer, RIG-I-like receptors, TLR

## Abstract

The innate immune sensing system serves as a critical line of defense for recognizing tumor-associated abnormal nucleic acids and initiating antitumor immune responses, and it plays an essential role in lung cancer development, progression, and therapeutic response. Recent studies have shown that, under conditions such as genomic instability, mitochondrial damage, epigenetic dysregulation, and therapy-induced stress, lung cancer cells can release a variety of abnormal DNA, RNA, and damage-associated molecular patterns (DAMPs), thereby activating key innate immune pathways, including cGAS–STING, Toll-like receptor (TLR), and RIG-I-like receptor (RLR) signaling. These pathways regulate the recruitment, activation, and functional differentiation of multiple immune cell populations, including dendritic cells, natural killer cells, neutrophils, macrophages, and T cells, through the induction of type I interferons, inflammatory cytokines, and chemokines, thereby shaping an antitumor immune network in lung cancer. However, during long-term evolution, lung cancer develops multilayered immunosuppressive mechanisms that inhibit innate immune signaling through genetic alterations, epigenetic modifications, ubiquitin-mediated protein degradation, metabolic reprogramming, and non-coding RNA regulation. These processes impair antigen presentation, restrict effector lymphocyte infiltration, and promote the accumulation of immunosuppressive cell populations such as tumor-associated macrophages, myeloid-derived suppressor cells, and regulatory T cells, ultimately leading to immune evasion. This review comprehensively discusses the activation mechanisms of three major innate immune pathways in lung cancer—cGAS–STING, TLR, and RLR signaling—the tumor-mediated negative regulatory mechanisms that suppress them, and their impact on the tumor immune microenvironment. Furthermore, it discusses the potential clinical value of targeting these pathways in immunotherapy, with the aim of providing a theoretical basis for optimizing lung cancer immunotherapeutic strategies and designing combination interventions.

## 1 Introduction

Lung cancer is one of the most frequently diagnosed and deadliest malignancies worldwide, posing a major threat to public health. According to epidemiological statistics, lung cancer has long ranked first among the leading causes of cancer-related mortality globally (1). Histologically, lung cancer is broadly classified into two major subtypes: non-small cell lung cancer (NSCLC) and small cell lung cancer (SCLC) (2, 3), with NSCLC accounting for approximately 85% of all cases (1, 4). In recent years, the widespread application of molecular targeted therapies and immune checkpoint inhibitors (ICIs) has significantly improved survival in a subset of patients. However, due to tumor molecular heterogeneity, the immunosuppressive tumor microenvironment, and the emergence of acquired resistance, a substantial proportion of patients still show limited responses to current therapeutic strategies (5–7). Therefore, a deeper understanding of the molecular mechanisms underlying immune regulatory networks during lung cancer initiation and progression is of great importance for optimizing immunotherapeutic approaches.

In antitumor immunity, the innate immune system constitutes the first line of defense for recognizing abnormal signals and initiating immune responses. Through a variety of pattern recognition receptors (PRRs), this system senses abnormal nucleic acids and damage-associated molecular patterns (DAMPs) derived from pathogens or tumor cells, thereby inducing the production of type I interferons (IFN-I), inflammatory cytokines, and chemokines, and promoting the recruitment and activation of immune cells such as dendritic cells (DCs), natural killer (NK) cells, and T cells (8, 9). Among these pathways, the cytosolic DNA-sensing cGAS–STING pathway, endosomal nucleic acid-sensing Toll-like receptors (TLRs), and cytosolic RNA-sensing RIG-I-like receptors (RLRs) represent the three major innate immune signaling axes in tumor immunity. By recognizing aberrant nucleic acids and inducing IFN-I responses, these pathways play central roles in bridging innate and adaptive immunity (10–12).

Despite their critical roles in antitumor immune surveillance, innate immune pathways can be actively suppressed by tumor cells during long-term evolution, thereby facilitating immune evasion. Rather than simply escaping immune recognition, tumor immune evasion is generally considered an active and dynamic process in which tumor cells, under the selective pressure of immunoediting, continuously reshape their interactions with the host immune system through acquired molecular alterations (13, 14). Tumor cells may inhibit antigen presentation, impair effector immune cell functions, and remodel the tumor immune microenvironment through mechanisms such as genetic mutations, epigenetic modifications, metabolic reprogramming, and non-coding RNA-mediated regulation (15–17). Meanwhile, immunosuppressive cell populations, including tumor-associated macrophages (TAMs), myeloid-derived suppressor cells (MDSCs), and regulatory T cells (Tregs), accumulate within the tumor microenvironment and further suppress the antitumor activity of T cells and NK cells, ultimately establishing an immunosuppressive network that favors tumor growth, metastasis, and therapeutic resistance (18–21). Therefore, a comprehensive review of the activation mechanisms of innate immune pathways in lung cancer, the tumor-mediated negative regulatory mechanisms that restrain them, and their impact on the tumor immune microenvironment will be of great significance for understanding immune remodeling in lung cancer and for exploring novel combination immunotherapeutic strategies.

## 2 Mechanisms by which negative regulators antagonize the cGAS–STING pathway in lung cancer immune evasion

### 2.1 Cytosolic DNA-driven activation of the cGAS–STING pathway in lung cancer

**Figure 1:**
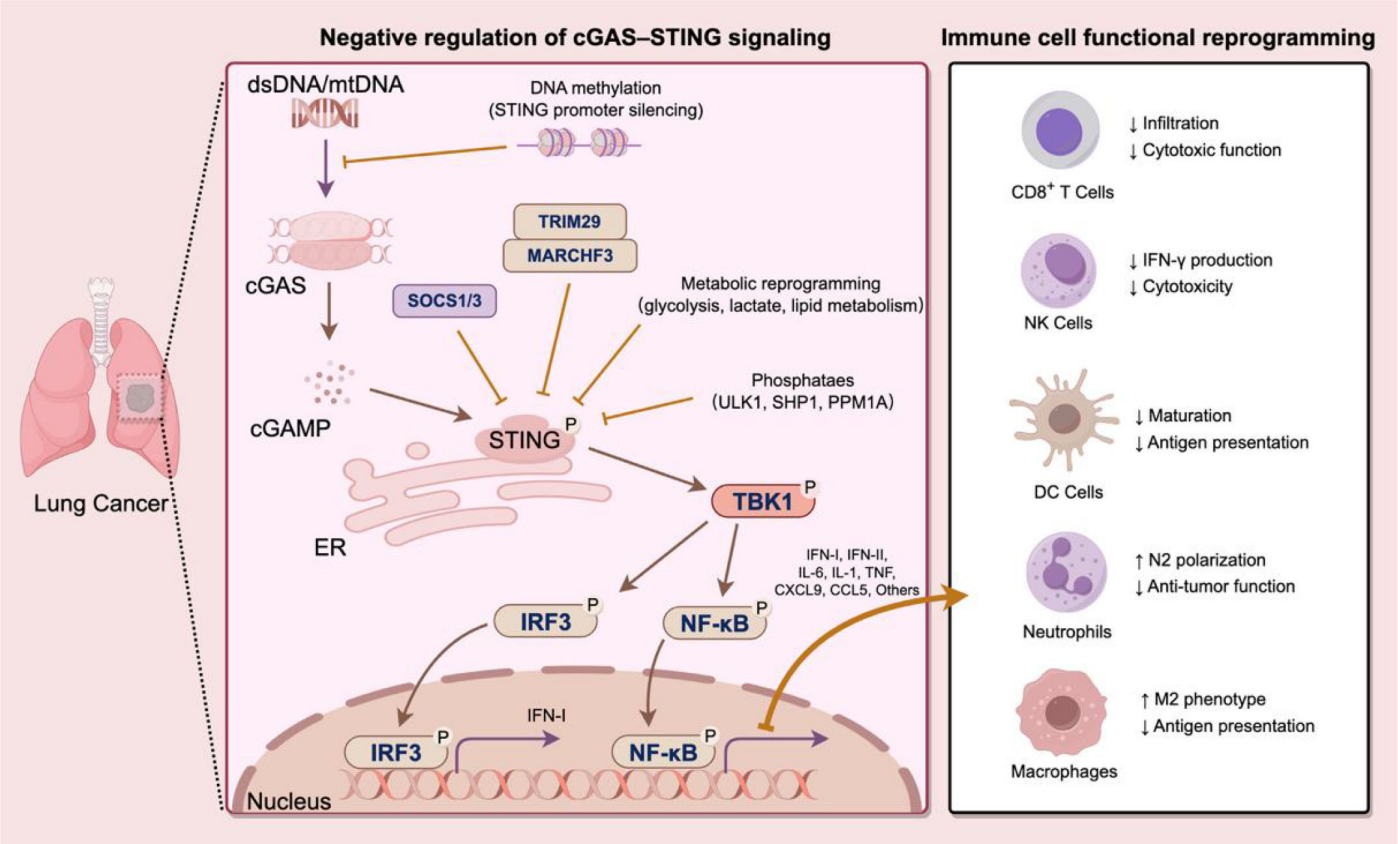
Negative regulation of cGAS–STING signaling drives immune cell functional reprogramming in lung cancer. In lung cancer, cytosolic DNA activates the cGAS–STING pathway, but its signaling is frequently suppressed by multiple mechanisms, including STING promoter methylation, TET2 loss, ubiquitin-mediated degradation (TRIM29, MARCHF3), phosphatase activity (ULK1, SHP1, PPM1A), and metabolic reprogramming. In addition, SOCS1/3-mediated inhibition further attenuates downstream signaling. As a result, IRF3-dependent type I interferon (IFN-I) and chemokine production (CXCL9, CXCL10, CCL5) are reduced, whereas NF-κB signaling remains partially active, promoting inflammatory cytokine production. This imbalance leads to impaired dendritic cell activation, reduced CD8^+^ T cell and NK cell function, and expansion of immunosuppressive cells, including MDSCs, M2-like macrophages, Tregs, and N2 neutrophils, thereby facilitating tumor immune evasion and resistance to immunotherapy.

Under physiological conditions, the cytoplasm is normally devoid of free double-stranded DNA (dsDNA), and its presence is regarded as a danger signal that activates innate immunity. In lung cancer cells, genomic instability, organelle damage, and therapy-induced stress lead to the release of DNA fragments from multiple sources into the cytoplasm. Cyclic GMP-AMP synthase (cGAS), a cytosolic DNA sensor, binds dsDNA in a sequence-independent manner and catalyzes the production of 2′3′-cGAMP, which subsequently activates STING located on the endoplasmic reticulum (8, 22, 23). Activated STING translocates to the Golgi apparatus, recruits TANK-binding kinase 1 (TBK1), and promotes the phosphorylation and nuclear translocation of interferon regulatory factor 3 (IRF3), thereby inducing IFN-I expression. At the same time, STING also activates the nuclear factor kappa-B (NF-κB) pathway to drive the production of inflammatory cytokines (24–28). As illustrated in **Figure 1**, cGAS–STING signaling in lung cancer links cytosolic DNA sensing to IRF3/NF-κB activation and subsequent immune remodeling.

In NSCLC, cytosolic DNA arises from highly diverse sources. With regard to nuclear genomic DNA, chromosomal instability (CIN)-associated mitotic errors can generate micronuclei or chromatin bridges, and rupture of their nuclear envelope exposes genomic DNA to the cytoplasm, where it is recognized by cGAS (29–31). Replication stress, defects in DNA repair, and abnormalities in damage response pathways such as ATM can also lead to DNA leakage into the cytosol (32–36). In addition, extrachromosomal DNA (ecDNA) and telomere-associated DNA may also serve as cGAS ligands and are closely associated with tumor aggressiveness and therapeutic resistance (37–39).

In addition to nuclear DNA, mitochondrial DNA (mtDNA) represents another major source of cytosolic DNA. Because mtDNA lacks histone protection and is relatively hypomethylated, it is more prone to cytosolic release under conditions of oxidative stress or mitochondrial dysfunction. Oxidatively modified mtDNA, such as 8-oxodG-containing species, exhibits enhanced cGAS-binding capacity (40–46). Moderate mtDNA signaling may facilitate antigen presentation and activate antitumor immunity, whereas persistent low-level mtDNA release may sustain chronic inflammation through NF-κB signaling and thereby promote tumor progression (47–49).

Furthermore, endogenous retroelements (EREs) can be reactivated in the context of DNA hypomethylation in lung cancer. Retrotranscription products derived from transposable elements such as LINE-1 and endogenous retroviruses (ERVs) can generate cDNA and induce a state of “viral mimicry,” thereby activating STING signaling (50–53). In addition, some ERV-encoded proteins may serve as tumor antigens that are presented by major histocompatibility complex class I (MHC-I) molecules, thereby enhancing immune surveillance (54, 55). Importantly, the biological output of cGAS–STING activation in lung cancer is not uniform. Rather, it is shaped by the cellular compartment in which signaling occurs, the amplitude and duration of activation, subcellular trafficking of pathway components, and crosstalk with metabolic, autophagic, senescence-associated, and inflammatory programs. Thus, acute robust activation may favor IFN-I-dominant antitumor immunity, whereas persistent low-level signaling may instead sustain chronic inflammation, adaptive stress responses, and tumor-promoting remodeling of the microenvironment (27, 29, 56–58). Moreover, the metal ion Mn²^+^ can increase cGAS sensitivity to dsDNA and further amplify signaling responses (59).

### 2.2 Molecular mechanisms underlying negative regulation of the cGAS–STING pathway in lung cancer

Although the cGAS–STING pathway is an important mediator of antitumor immunity, its functional outputs in lung cancer are not strictly linear and may vary according to cellular context. Thus, the consequences of negative regulation should not be viewed as uniformly equivalent across tumor cells and immune cells. Nevertheless, in many lung cancer settings, tumor-intrinsic and microenvironmental suppressors converge to attenuate beneficial innate immune priming and thereby promote immune evasion.

At the epigenetic level, lung cancer cells can inhibit STING expression through DNA methylation. For example, DNA methylation within the STING promoter region can markedly reduce its transcriptional activity, thereby decreasing IFN-I production and DC antigen presentation (60). In addition, genetic mutations and epigenetic alterations may also disrupt cGAS–STING signaling. Loss of STK11/LKB1, which frequently co-occurs with KRAS mutations, can significantly suppress STING pathway activity (61–63). Likewise, reduced TET2 expression and decreased levels of 5-hydroxymethylcytosine (5-hmC) are also associated with impaired DNA sensing (64).

At the protein regulatory level, multiple factors have been reported to suppress STING signaling through post-translational modifications. Phosphatases such as ULK1, PPM1A, and SHP1 can inhibit STING activation through dephosphorylation (65, 66). In addition, E3 ubiquitin ligases including TRIM29 have been implicated in negative regulation of STING signaling (67), whereas other regulators such as MARCHF3 have been reported to modulate STING stability in non-lung cancer settings (68). These findings suggest that ubiquitin-mediated control of STING may also contribute to signal attenuation in lung cancer, although direct evidence for some regulators remains limited.

Recent studies have also shown that STING functions as a metabolic checkpoint in tumor cells. STING signaling can restrict aerobic glycolysis by inhibiting hexokinase 2 (HK2) activity, whereas suppression of the STING pathway facilitates glucose uptake and glycolytic flux, thereby promoting lactate production and meeting the energetic and biosynthetic demands of rapid proliferation (69). In addition, STING inactivation is associated with mitochondrial homeostasis imbalance and lipid metabolic reprogramming, which can promote lipid synthesis and fatty acid utilization, thereby maintaining redox homeostasis and conferring survival advantages to tumor cells (70, 71). These metabolic changes not only support tumor cell growth but also reshape the tumor microenvironment through lactate accumulation and lipid remodeling, thereby promoting the expansion of immunosuppressive cell populations such as MDSCs, TAMs, Tregs (72–75). Thus, lung cancer persistently suppresses the cGAS–STING signaling axis through epigenetic silencing, post-translational protein regulation, and metabolic reprogramming, thereby attenuating IFN-I responses and creating favorable conditions for immune evasion.

Beyond tumor cell-intrinsic epigenetic silencing and post-translational modifications, suppressive factors within the immune microenvironment also contribute to the inactivation of the cGAS–STING pathway. Among these, TRIM family proteins play a crucial role in the negative regulation of innate immunity. Notably, TRIM29 may represent a particularly relevant suppressor in lung cancer. Previous mechanistic studies have shown that TRIM29 restricts DNA-induced type I interferon responses by promoting ubiquitin-mediated degradation of STING. Importantly, independent studies in lung cancer have linked TRIM29 to tumor progression, metastasis, and therapeutic resistance (76–78). These observations suggest that TRIM29 may function as a bridge regulator linking tumor progression with suppression of STING-mediated antitumor immunity in the lung tumor microenvironment (67, 79). Meanwhile, suppressor of cytokine signaling 1 (SOCS1) and SOCS3, as classical negative feedback regulators of cytokine signaling, can inhibit Janus kinase/signal transducer and activator of transcription (JAK–STAT) signaling and IFN-associated amplification loops, thereby weakening STING downstream IFN-I output and limiting the expansion of antitumor immunity (80, 81). The major inhibitory mechanisms discussed above, together with their downstream effects on immune-cell function, are summarized in **Figure 1**.

### 2.3 Context-dependent impact of cGAS–STING attenuation on the tumor microenvironment

As shown in **Figure 1**, attenuation of STING signaling in lung cancer often contributes to an immunosuppressive microenvironment; however, this effect is context dependent rather than strictly linear. The consequences of cGAS–STING signaling differ between tumor cells and host immune cells, and are further shaped by disease stage, anatomical site, and signaling kinetics (58). In particular, STING activation in dendritic cells is closely linked to type I interferon production, antigen cross-presentation, and T-cell priming (82, 83), whereas tumor-cell-intrinsic STING may generate either immunostimulatory or tumor-supportive outputs depending on signal strength and persistence (84, 85). Thus, although attenuation of this pathway may not exert identical effects across all cellular compartments, in many lung cancer settings it predominantly impairs productive innate immune priming and favors immune evasion.

During the initiation of antitumor immunity, DCs are among the key immune populations regulated by STING signaling. Under physiological conditions, tumor-derived DNA can be taken up by DCs and activate the cGAS–STING pathway, thereby inducing IFN-I production and promoting DC maturation. This process enhances the expression of major histocompatibility complex class I (MHC-I) and costimulatory molecules such as CD40, CD80, and CD86, ultimately improving the efficiency of antigen cross-presentation (10, 82, 86, 87). In addition, cGAMP produced by tumor cells can be transferred intercellularly to activate tumor-infiltrating DCs, thereby amplifying STING-dependent immune responses (88). Accordingly, attenuation of STING signaling is generally associated with impaired DC maturation and reduced antigen presentation efficiency, which weakens the priming and expansion of naïve T cells (82, 89, 90).

STING pathway impairment also affects NK cell-mediated innate immune responses. STING-induced IFN-I and IL-15 are important for maintaining NK cell survival and effector function, as they promote IFN-γ secretion and cytotoxic activity (83, 91). In many lung cancer contexts, suppression of STING signaling is associated with reduced NK cell recruitment and activation and may contribute to functional exhaustion, thereby weakening NK cell-mediated tumor elimination (92). In addition, defective STING signaling has been associated with reduced immune surveillance in metastatic lung adenocarcinoma lesions, potentially increasing the risk of tumor recurrence (93).

In myeloid immune cells, loss of STING signaling also alters the polarization states of neutrophils and macrophages. Neutrophils are highly plastic and can be categorized into antitumor N1 and protumor N2 phenotypes, with IFN-I considered an important signal for maintaining the N1 phenotype (94, 95). When the STING pathway is suppressed, reduced IFN-I levels can drive tumor-associated neutrophils toward the protumor N2 phenotype and enhance their immunosuppressive and tumor-promoting functions (96, 97). Similarly, STING deficiency weakens the pro-inflammatory response of macrophages, making them more likely to polarize toward the immunosuppressive M2 phenotype, thereby reducing antigen presentation capacity and promoting tumor progression (98–100).

At the adaptive immune level, suppression of the STING pathway significantly reduces the infiltration of CD8^+^ cytotoxic T lymphocytes (CTLs) into tumors. STING-dependent chemokines, such as CXCL9, CXCL10, and CCL5, establish chemotactic gradients within tumor tissues and promote CTL recruitment (87). Therefore, when STING signaling is impaired, the number of CTLs in the tumor microenvironment declines, resulting in weakened antitumor immune responses (101–103). Clinical studies have further shown that tumors lacking STING signaling typically exhibit lower levels of T-cell infiltration and are closely associated with resistance to immune checkpoint inhibitor therapy.

## 3 Mechanisms by which negative regulators antagonize the TLR pathway in lung cancer immune evasion

### 3.1 Activation of the TLR pathway in lung cancer

**Figure 2:**
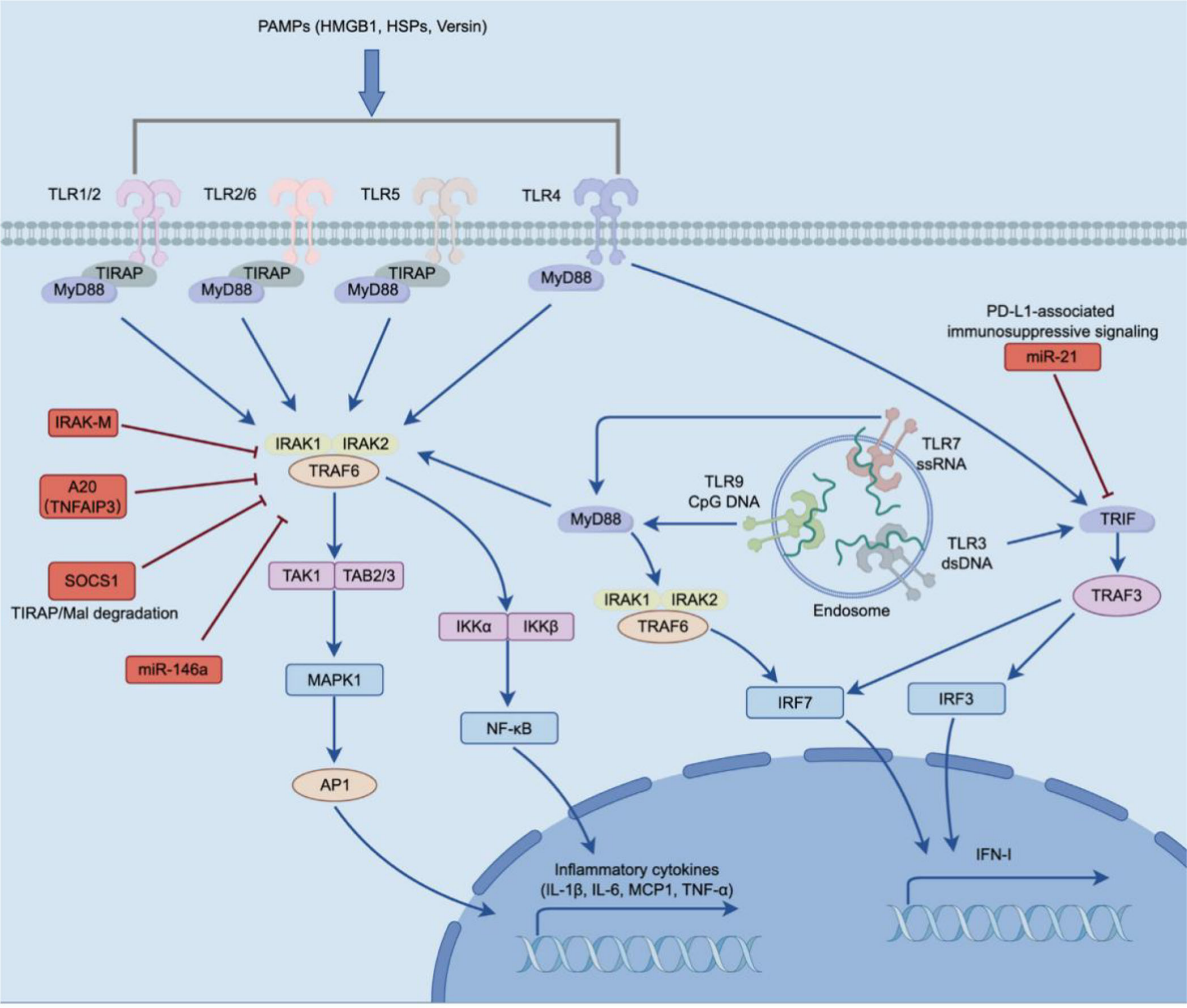
Negative regulation of TLR signaling in lung cancer. In lung cancer, TLR signaling is activated by extracellular and endosomal danger signals, but its effective antitumor output is frequently attenuated by multiple negative regulatory mechanisms. At the proximal signaling level, IRAK-M suppresses MyD88-dependent signaling, A20 (TNFAIP3) disrupts TRAF6-associated signal propagation, and SOCS1 promotes TIRAP/Mal degradation, thereby limiting downstream NF-κB and MAPK activation. In parallel, miR-146a targets IRAK1 and TRAF6 to form a negative feedback loop, whereas miR-21 is associated with PD-L1-related immunosuppressive signaling. Endosomal TLR3/7/9 and surface TLR4 can also engage TRIF-dependent signaling; however, persistent inhibitory inputs dampen IRF3/IRF7-mediated type I interferon production. Collectively, these regulatory networks shift TLR signaling away from effective antitumor immunity toward chronic inflammatory and immunosuppressive outputs in the lung tumor microenvironment.

In the lung cancer microenvironment, the activation of TLRs primarily originates from the recognition of pathogen-associated molecular patterns (PAMPs) and DAMPs, triggering complex and heterogeneous signal transduction processes in tumor cells, immune cells, and stromal cells (104, 105). TLRs are type I transmembrane proteins containing an extracellular leucine-rich repeat (LRR) ligand recognition domain and an intracellular Toll/interleukin-1 receptor (TIR) domain (105). The human TLR family consists of 10 members, which can be divided into cell surface receptors (TLR1, TLR2, TLR4, TLR5, TLR6, and TLR10) and endosomal receptors (TLR3, TLR7, TLR8, and TLR9) based on their subcellular localization (106, 107). At the signal transduction level, except for TLR3, most TLRs rely on myeloid differentiation primary response 88 (MyD88) to recruit the IRAK family and TRAF6, thereby activating the NF-κB and MAPK pathways; TLR3 activates IRF3 and induces IFN-I production via TRIF, while TLR4 possesses the dual-pathway characteristics of both MyD88 and TRIF (108). The dynamic balance of these signaling axes largely determines whether the lung cancer microenvironment tends toward anti-tumor immune activation or the formation of a pro-tumorigenic chronic inflammatory state (108). This overall signaling framework and its shift toward immunosuppressive outputs are summarized in **Figure 2**. Importantly, the biological consequences of TLR activation in lung cancer are highly context dependent and may vary according to receptor subtype, cellular compartment, disease stage, and the intensity and duration of stimulation. In particular, acute TLR activation in professional immune cells may enhance antigen presentation and antitumor immunity, whereas persistent low-level stimulation in tumor cells or myeloid cells may instead favor chronic inflammation, immune suppression, and tumor adaptation (109, 110).

In lung cancer tissues, nucleic acid-sensing TLRs primarily detect abnormal DNA or RNA signals. TLR3 recognizes double-stranded RNA (dsRNA). Its endogenous ligands include exosomes rich in non-coding RNA and dsRNA fragments released by lung cancer cells; after sensing these signals via TLR3, lung epithelial cells can activate TRIF-dependent pathways and induce chemokine secretion, thereby promoting neutrophil recruitment and pre-metastatic niche formation (111, 112). The exogenous TLR3 agonist Poly(I:C) can directly induce tumor cell apoptosis and upregulate MHC-I expression, thereby enhancing T cell-mediated anti-tumor immune surveillance (113). TLR7/8 primarily recognize guanosine- and uridine-rich single-stranded RNA (ssRNA). RNA generated by highly active LINE-1 retrotransposons in lung cancer cells can serve as endogenous ligands to activate TLR7/8 and upregulate the anti-apoptotic protein Bcl-2 via the non-canonical NF-κB pathway, enhancing chemotherapy tolerance (114). TLR9 recognizes unmethylated CpG DNA. Mitochondrial DNA and DNA fragments released by apoptosis, necrosis, or genomic instability in the lung cancer microenvironment can all serve as potential ligands (115). TLR9 activation also has dual effects: in plasmacytoid DCs, it can induce IFN-α production and enhance anti-tumor immunity, whereas in tumor cells, it may promote invasiveness and anti-apoptotic signaling (116).

In addition to nucleic acid-sensing receptors, membrane surface TLRs also play important roles in lung cancer-associated inflammation. TLR2 can form heterodimers with TLR1 or TLR6 to expand its range of ligand recognition (117), while the proteoglycan Versican, generated by extracellular matrix remodeling, can act as a key endogenous DAMP to activate the TLR2/TLR6 complex on the surface of tumor-associated macrophages. This induces the secretion of pro-inflammatory cytokines such as TNF-α, IL-6, and CCL2, driving the polarization of macrophages toward an immunosuppressive M2 phenotype and promoting tumor invasion and metastasis (118, 119). Furthermore, TLR4 can recognize not only lipopolysaccharide (LPS) but also respond to endogenous DAMPs such as HMGB1; especially during cell death induced by chemotherapy or radiotherapy, HMGB1 is released in large quantities and simultaneously activates the MyD88 and TRIF signaling axes via TLR4, further regulating chemokine secretion and the functional state of DCs (120). Clinical studies have also shown that high TLR4 expression is generally associated with a poorer prognosis in lung cancer patients (121).

### 3.2 Immune evasion mechanisms mediated by negative regulation of the TLR pathway in lung cancer

Although the TLR pathway can sense danger signals and initiate antitumor immunity, its biological outputs in lung cancer are not uniformly protective and are shaped by receptor subtype, cell type, disease stage, and signaling kinetics. In this context, lung cancer cells can remodel TLR signaling into a protumorigenic and immunosuppressive axis through persistent low-intensity stimulation and multilayered molecular regulation during long-term evolution (105, 122). Lung cancer cells continuously release DAMPs such as HMGB1, HSPs, and nucleic acid fragments during rapid proliferation, necrosis, or treatment-induced damage. These molecules can repeatedly stimulate the TLR pathway, induce chronic inflammatory responses, and promote the production of immunosuppressive cytokines such as IL-6 and IL-10, thereby gradually forming an inflammatory microenvironment conducive to tumor growth (123–126). Therefore, in lung cancer, against the backdrop of continuous low-level stimulation, TLR signaling can gradually shift from an acute anti-tumor immune response to a pro-tumorigenic chronic inflammatory and immunosuppressive state (123, 127). This transition is unlikely to occur identically across all cellular compartments; rather, TLR signaling may exert divergent effects in tumor cells, dendritic cells, macrophages, and other stromal or immune populations.

At the molecular level, TLR signaling is inhibited by negative regulatory factors at multiple key nodes. As illustrated in **Figure 2**, these inhibitory inputs converge on both MyD88-dependent and TRIF-associated branches, thereby attenuating effective antitumor signaling. IRAK-M (IRAK3) is an important inhibitory protein of the MyD88 complex. It can inhibit downstream NF-κB signaling activation by stabilizing the Myddosome and preventing the dissociation of IRAK1 and IRAK4, while simultaneously promoting the polarization of tumor-associated macrophages toward the immunosuppressive M2 phenotype (128–130). A20 (TNFAIP3) is a ubiquitin-editing enzyme and classical negative regulator of TLR/NF-κB signaling that suppresses pathway activation by removing K63-linked polyubiquitin chains from TRAF6 and related adaptor complexes. Emerging evidence suggests that A20 also has context-dependent roles in lung cancer, where reduced expression has been associated with immune evasion and impaired CD8^+^ T-cell surveillance. In addition, A20 may regulate the TBK1–STAT1–PD-L1 axis and influence responses to immune checkpoint blockade (131, 132). In addition, SOCS1 can bind to the adaptor protein Mal/TIRAP and promote its ubiquitin-mediated degradation, further inhibiting the TLR cascade (133–135). These negative regulatory mechanisms collectively attenuate TLR-mediated inflammatory responses and IFN-I production, causing the lung cancer microenvironment to gradually shift toward an immunosuppressive state (136).

Beyond protein-level regulation, lung cancer can also perform epigenetic remodeling of the TLR pathway through non-coding RNA networks. miR-146a can form a classic negative feedback loop for TLR signaling by targeting IRAK1 and TRAF6, while miR-146a from lung cancer cell-derived exosomes can also reduce the responsiveness of monocytes and macrophages to TLR stimulation (137, 138). In contrast, miR-21 is frequently overexpressed in lung cancer and may contribute to TLR-associated protumor and immunosuppressive signaling through both intracellular and intercellular mechanisms (139). In primary human lung cancer cells, LPS-induced TLR4 activation has been shown to upregulate miR-21 via a ROS-dependent pathway, and silencing miR-21 attenuates the tumor-promoting effect of TLR4 signaling (140). In addition, miR-21 can enhance oncogenic signaling through the PTEN/PI3K/AKT axis, and tumor-derived extracellular miR-21 has been shown in broader cancer models to act as a paracrine ligand for TLR7/8 on immune cells (141, 142), thereby triggering prometastatic inflammatory responses and potentially reinforcing an immune-evasive microenvironment in lung cancer. Furthermore, lncRNAs are also involved in TLR-associated immunosuppression; for example, XIST upregulates PD-L1 expression by downregulating miR-34a-5p, while MIR17HG promotes Treg expansion by inhibiting RUNX3 (143–145). Thus, through continuous inflammatory stimulation, negative regulatory proteins, and non-coding RNA networks, lung cancer collectively suppresses the effective anti-tumor output of TLR signaling and lays the foundation for immune evasion (138, 145–147).

Additionally, SOCS family molecules in the immune microenvironment are important auxiliary inhibitory factors for the TLR pathway. SOCS1 and SOCS3 can not only inhibit JAK-STAT-dependent inflammatory amplification responses but also directly suppress TLR7-mediated IFN-I production by promoting the degradation of interferon regulatory factor 7 (IRF7), thereby limiting the anti-tumor immune activation of DCs and myeloid cells (148, 149).

### 3.3 Impact of impaired TLR pathway on the tumor microenvironment

The consequences of TLR pathway imbalance in the lung cancer microenvironment are context dependent rather than strictly uniform. Under normal conditions, appropriately activated TLR signaling can promote dendritic cell maturation, enhance antigen presentation, and support effector lymphocyte activation. However, under conditions of chronic stimulation, pathway reprogramming, or selective suppression of immunostimulatory branches, these protective effects may be progressively replaced by protumor inflammation and immune suppression. Thus, although impaired or maladaptively rewired TLR signaling does not exert identical effects across all receptor subtypes and cellular compartments, in many lung cancer settings it contributes overall to defective immune priming and tumor immune evasion.

First, DCs often remain in an immature state due to tumor-derived suppressive factors. Endosome-localized TLR7/8/9 can originally upregulate costimulatory molecules such as CD80, CD86, and CD40 via MyD88–NF-κB, promoting DC maturation and enhancing antigen cross-presentation (150). However, in lung cancer, although the DC3 subpopulation can expand and become activated under TLR9 stimulation, this is often accompanied by the compensatory upregulation of PD-L1, thereby limiting sustained T cell activation (151). Correspondingly, combining TLR9 activation with PD-L1 inhibition can significantly increase the number and cytotoxic activity of tumor-infiltrating T cells (152), suggesting that TLR functional imbalance directly impairs antigen presentation and T cell priming.

Second, impaired TLR pathways also affect the effector functions of NK cells and CD8^+^ T cells. NK cells express TLR3 and can directly sense dsRNA to enhance cytotoxic activity (153, 154); meanwhile, TLR7/8 and TLR9 activation can also promote NK cell IFN-γ secretion and killing responses through the “bystander effect” of IL-12, IL-18, and IFN-I (155–157). Therefore, when TLR signaling is continuously suppressed or skewed toward protumor outputs, NK cell cytotoxicity is often weakened in lung cancer-associated microenvironments. Similarly, TLR signaling plays an important role in CD8^+^ T cell anti-tumor immunity: TLR2 deficiency can impair T cell costimulatory capacity (158). Conversely, the activation of TLR3, TLR4, TLR7, and TLR9 all contribute to enhancing tumor-specific CD8^+^ T cell infiltration and cytotoxic function (113, 159–161). Accordingly, impairment or maladaptive rewiring of TLR signaling may reduce effector T-cell recruitment and weaken adaptive antitumor immunity.

In myeloid immune cells, TLR signaling imbalance can also drive the shift of neutrophils and macrophages toward pro-tumor phenotypes. Normally, a TLR/IFN-β background helps maintain anti-tumor N1 neutrophils, characterized by the upregulation of CCL3, CXCL9, and CXCL10, as well as enhanced ROS- and TRAIL-dependent cytotoxicity (94, 162, 163). However, in an environment dominated by TGF-β and IL-10, neutrophils are more prone to shift toward the N2 phenotype, suppressing T cells and promoting metastasis by depleting L-arginine via ARG1, releasing neutrophil elastase (NE), and forming neutrophil extracellular traps (NETs) (95, 164–172). At the same time, TLR activation in macrophages could originally drive their transformation toward an M1-like anti-tumor phenotype, enhancing phagocytosis and T cell recruitment capabilities; but when TLR signaling is suppressed or reprogrammed by the tumor, macrophages are more likely to maintain an immunosuppressive M2 phenotype, thereby further promoting tumor growth and immune evasion (173–178). Overall, impairment of the TLR pathway not only weakens antigen presentation and effector lymphocyte activation but also drives the lung cancer microenvironment to transition into a stable immunosuppressive state by reshaping the composition and function of myeloid cells.

## 4 Mechanisms by which negative regulators antagonize the RLR pathway in lung cancer immune evasion

### 4.1 Activation of the RLR pathway in lung cancer

**Figure 3:**
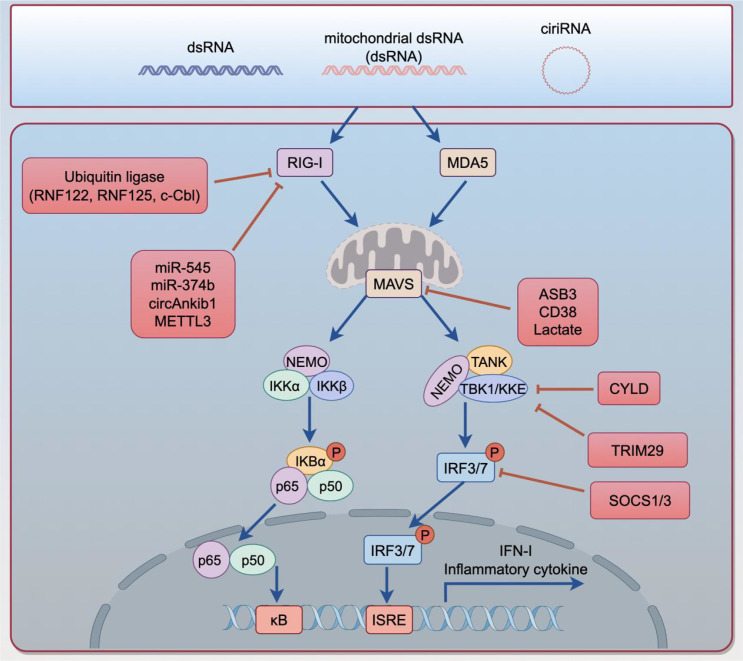
Negative regulation of RLR signaling in lung cancer. In lung cancer, abnormal endogenous RNAs, including cytosolic dsRNA, mitochondrial dsRNA, and immunostimulatory circular RNAs, can activate the RLR pathway through RIG-I and MDA5. Upon RNA sensing, RIG-I/MDA5 signal through mitochondrial MAVS, leading to activation of the NEMO–IKKα/IKKβ–NF-κB axis and the TANK–TBK1/IKKϵ–IRF3/7 axis, thereby inducing type I interferon (IFN-I) and inflammatory cytokine expression. However, this pathway is frequently attenuated by multiple negative regulatory mechanisms in lung cancer. E3 ubiquitin ligases, including RNF122, RNF125, and c-Cbl, promote ubiquitin-dependent degradation of RIG-I; miR-545, miR-374b, circAnkib1, and METTL3 suppress RLR signaling at the RNA or epigenetic level; ASB3, CD38, and lactate impair MAVS stability or signaling activity; CYLD dampens downstream signaling by removing activating ubiquitin chains; and SOCS1, SOCS3, and TRIM family proteins further restrict IRF3/7-dependent interferon responses. Collectively, these inhibitory mechanisms weaken antiviral-like innate immune signaling and facilitate tumor immune evasion in lung cancer.

RLRs are cytosol-localized RNA sensors, primarily including RIG-I (DDX58) and MDA5, which trigger antiviral-like innate immune responses by recognizing abnormal RNA (179). RIG-I consists of N-terminal tandem CARD domains, a central DExD/H-box helicase domain, and a C-terminal domain, and is capable of recognizing short dsRNA or stem-loop RNA structures bearing a 5′-triphosphate end (180, 181). In its resting state, RIG-I exists in an autoinhibited conformation. Upon binding to RNA ligands, an ATP-dependent conformational change releases the CARD domains and promotes K63-linked ubiquitination, thereby enhancing receptor aggregation and signal transduction (182, 183). Activated RIG-I subsequently binds to the mitochondrial antiviral-signaling protein (MAVS) on the outer mitochondrial membrane, inducing its prion-like polymerization and activating the TBK1-IRF3/7 and NF-κB signaling pathways, which in turn induce the expression of IFN-I and inflammatory cytokines (184, 185). As illustrated in **Figure 3**, this RNA-sensing cascade forms the central RLR signaling axis that is subsequently targeted by multiple negative regulators in lung cancer. In the context of tumors, this pathway is often triggered by endogenous RNA to form a state of “viral mimicry,” thereby enhancing tumor immunogenicity and influencing responses to immune checkpoint therapy (52, 186). However, the biological consequences of RLR activation in lung cancer are also context dependent and may differ according to cellular compartment, tumor stage, and signaling dynamics. Acute activation in antigen-presenting cells or tumor cells may promote IFN-I-dominant antitumor immunity, whereas persistent low-level signaling or chronic interferon exposure may instead support adaptive resistance, immune exhaustion, or therapy tolerance.

In lung cancer, various abnormal RNAs can serve as RLR ligands. First, epigenetic imbalance can lead to the reactivation of transposable elements such as ERVs, forming dsRNA through bidirectional transcription and inducing an IFN-I response (50, 51). In small cell lung cancer, the expression of long terminal repeats such as LTR30 and LTR9C is significantly correlated with RIG-I and innate immune gene signatures, where high LTR30 expression is often accompanied by a stronger IFN-I response (187). Furthermore, ADAR1 deficiency can lead to the accumulation of endogenous dsRNA and activate the RIG-I/MDA5-MAVS axis, thereby enhancing anti-tumor immunity and inhibiting tumor growth (188).

In addition to ERV-derived RNA, mitochondria-derived double-stranded RNA (mtdsRNA) is also an important RLR ligand. Normally, these RNAs are cleared by mitochondrial RNA degradation systems such as PNPase and SUV3 (189). However, in non-small cell lung cancer, the imbalance of mitochondrial RNA homeostasis leads to the accumulation of mtdsRNA, which is released into the cytosol via VDAC1/2- or BAX/BAK-mediated mitochondrial membrane permeabilization, thereby activating IFN-I-related signaling (189, 190). Moreover, some non-coding RNAs can also directly activate RIG-I. For example, 7SL RNA, generated by excessive RNA polymerase III transcription driven by the NOTCH1-MYC axis, can be transferred to neighboring cells via exosomes and trigger RIG-I signaling (191); circular RNAs such as circNDUFB2 can also act as RIG-I ligands to participate in anti-tumor immune regulation (192). Notably, RNA polymerase III can also transcribe cytosolic DNA into 5′-triphosphate dsRNA, thereby mediating radiotherapy-induced anti-tumor immune responses via the Pol III–RIG-I–MAVS axis (193–195).

### 4.2 Immune evasion mechanisms mediated by negative regulation of the RIG-I pathway in lung cancer

Although the RLR pathway can sense abnormal RNA and initiate antitumor immunity, its downstream consequences in lung cancer are not uniformly beneficial and depend on cell type, signaling intensity, and temporal context. Nevertheless, lung cancer cells have evolved multilayered negative regulatory mechanisms that suppress this signaling axis and attenuate the beneficial components of the “viral mimicry” response.

At the level of protein homeostasis regulation, the ubiquitin-proteasome system is a critical mechanism for inhibiting RLR signaling (196, 197). Multiple E3 ubiquitin ligases can promote the degradation of RIG-I or MAVS via K48-linked ubiquitination (198–200). For example, RNF122 can target the RIG-I CARD domain and mediate its K48-linked ubiquitination at the K115 and K146 sites, thereby promoting proteasomal degradation; RNF125 can similarly mediate RIG-I degradation, forming a negative feedback regulation (198, 200). In addition, c-Cbl, in synergy with SIGLEC-G and SHP2, mediates K48-linked ubiquitination of RIG-I at the K813 site, while ASB3 catalyzes the formation of K48-linked ubiquitin chains on MAVS, leading to MAVS degradation and blocking TBK1-IRF3 signal transduction (199, 201). Through these mechanisms, lung cancer cells can terminate RLR signaling at the RNA sensing stage, thereby inhibiting IFN-I production.

Tumor metabolic reprogramming is also involved in RLR inhibition. The classical Warburg effect in lung cancer cells leads to the accumulation of lactate in the tumor microenvironment; lactate can directly bind to the transmembrane domain of MAVS and interfere with the interaction between RIG-I and MAVS, thereby blocking MAVS polymerization and the downstream signaling cascade (202). This mechanism suggests that tumor metabolites not only support proliferation but can also directly act as functional inhibitors of RLR signaling. Furthermore, non-coding RNAs and epigenetic regulation can also inhibit the RLR pathway. For example, miR-545 and miR-374b can target DDX58 (RIG-I) mRNA and reduce its expression (203–205); circular RNAs such as circAnkib1 can act as molecular decoys that bind directly to RIG-I, blocking its interaction with RNA ligands (203, 206). Conversely, circNDUFB2, which has an immune-activating effect, is often downregulated in lung cancer, thereby weakening RIG-I signaling (192). Concurrently, the m6A methyltransferase METTL3 can inhibit the RIG-I-MAVS signaling axis via m6A modification and promote tumor progression (207), whereas ADAR1 deficiency reactivates the RLR pathway through dsRNA accumulation (188)°.

Additionally, some molecules can directly interfere with core components of the RLR pathway. ORMDL3 can inhibit IFN-I signaling by promoting RIG-I degradation (208, 209), and CD38 inhibits interferon secretion and weakens CD8^+^ T cell anti-tumor immunity by inducing MAVS degradation (210). The deubiquitinating enzyme CYLD inhibits IRF3 signaling and interferon production by removing polyubiquitin chains from RIG-I and TBK1 (211). These regulatory mechanisms collectively form an important molecular network through which lung cancer suppresses the RLR pathway.

Beyond the intrinsic ubiquitination and degradation mechanisms of tumor cells, SOCS1/SOCS3 and TRIM family proteins may also serve as auxiliary inhibitory modules to further suppress RLR signaling. Among them, TRIM29 is of particular interest because it has been linked to lung cancer progression and may inhibit RNA-triggered interferon responses through targeting NEMO, MAVS, and TBK1-associated signaling, thereby extending the RLR inhibitory network beyond RNF122, c-Cbl, and ASB3 (212–214). SOCS1/SOCS3 can limit the JAK-STAT-dependent IFN amplification effect following RIG-I/MAVS activation. The TRIM family, a group of E3 ubiquitin ligases, is widely recognized as participating in the regulation of innate immune receptor stability and tumor immune evasion, suggesting they may act alongside RNF122, c-Cbl, and ASB3 to constitute the RLR inhibitory network (81, 215). The major inhibitory nodes acting at the levels of RIG-I, MAVS, and downstream interferon signaling are summarized in **Figure 3**.

### 4.3 Impact of impaired RLR pathway on the tumor microenvironment

As shown in **Figure 3**, impaired RLR signaling in lung cancer generally weakens IFN-I production, antigen presentation, and effector immune-cell recruitment, although these consequences remain context dependent rather than uniformly linear. In many settings, attenuation of the RIG-I–MAVS axis weakens IFN-I and chemokine production, thereby impairing antigen presentation, dendritic cell-mediated immune priming, and effector lymphocyte recruitment. However, the consequences of RLR signaling are also shaped by cellular compartment and signaling duration (216, 217). Acute RLR activation, including pharmacologic stimulation with RIG-I agonists, can enhance antitumor immunity and improve responsiveness to immune checkpoint blockade, whereas chronic low-level interferon signaling may induce adaptive resistance programs such as interferon-stimulated gene signatures associated with therapy tolerance (218). In addition, emerging evidence suggests that RIG-I may exert cell-intrinsic inhibitory effects in exhausted CD8^+^ T cells, further underscoring the non-linear nature of this pathway (219). Therefore, in the context of lung cancer immune evasion, suppression of the beneficial antitumor outputs of RLR signaling generally favors an immunosuppressive microenvironment, even though some branches of persistent signaling may themselves contribute to resistance or T-cell dysfunction.

At the level of innate immune priming, the antitumor arm of RLR signaling is particularly evident in tumor cells and antigen-presenting cells. In tumor cells, activation of the RIG-I–MAVS axis can enhance IFN-I-related immunogenic signaling and chemokine production, thereby facilitating immune recognition. In dendritic cells, during the initiation phase of antitumor immunity, recognition of 5′-ppp dsRNA by RIG-I induces the expression of IFN-I, CXCL9, and CXCL10 via the MAVS–TBK1–IRF3 pathway, thereby enhancing the antigen cross-presentation capacity of CD103^+^ cDC1s and promoting CD8^+^ T-cell infiltration (220). RIG-I agonists (such as 3pRNA) can significantly enhance DC maturation in lung cancer models and produce synergistic anti-tumor effects when combined with PD-1 blockade (221). In addition, RLR and STING signaling converge at the TBK1-IRF3 node and form a synergistic immune network mediated by IFN-I (222). However, these immunostimulatory effects are most evident in tumor cells and antigen-presenting cells and should not be directly extrapolated to all lymphocyte compartments.

Impairment of the RLR pathway also affects the functions of NK cells and CD8^+^ T cells. RIG-I signaling can promote the migration of NK cells to tumor tissues by inducing IFN-I and CXCL9/10/11 to form a CXCR3-dependent chemotactic axis (223, 224). Simultaneously, this pathway can induce the secretion of IL-12, IL-15, and IL-18, enhancing NK cell cytotoxicity and IFN-γ production (225–228). Furthermore, RIG-I can enhance TRAIL expression in NK cells and improve their ability to kill tumor cells (229). Therefore, when RLR signaling is impaired, NK cell recruitment and activation are diminished, and the capacity for early immune clearance of tumors declines. By contrast, the role of RIG-I in CD8^+^ T cells appears to be distinct from its generally immunostimulatory effects in dendritic cells and NK-associated immune circuits. RIG-I is highly expressed in tumor-infiltrating CD8^+^ T cells, particularly in the terminally exhausted subpopulation, and is considered to function similarly to an intracellular immune checkpoint, which can inhibit AKT/glycolysis and STAT5 signaling and promote T-cell apoptosis (230). Conversely, RIG-I deficiency can enhance CD8^+^ T cell survival and improve the anti-tumor efficacy of PD-1 blockade therapy (231). These findings suggest that the biological consequences of RLR signaling should be interpreted in a cell-type-specific manner rather than as a uniformly beneficial or uniformly suppressive pathway.

In myeloid immune cells, RLR signaling is also involved in regulating the functions of neutrophils and macrophages. IFN-I produced by MAVS signaling is a crucial condition for maintaining anti-tumor N1 neutrophils (232). Moreover, RIG-I activation can further enhance neutrophil immune surveillance by inducing immunogenic cell death and releasing DAMPs (97, 233–235). In macrophages, RLR signaling participates in the M1/M2 polarization program by regulating molecules such as ATF4 and SMAD4 (236). After activation, RIG-I can promote the transformation of macrophages toward an M1-like phenotype, enhance the expression of TNF-α, IL-1β, IL-6, and iNOS, and increase anti-tumor activity via the MAVS/NF-κB axis (236, 237). The RIG-I agonist SLR10 can also induce human alveolar macrophages to polarize toward the M1 phenotype, counteracting the immunosuppressive programs mediated by IL-9/Arg1 and TGF-β (238, 239). Therefore, when the RLR pathway is continuously suppressed, neutrophils and macrophages are more likely to maintain protumor and immunosuppressive phenotypes, thereby further favoring lung cancer immune evasion (240).

## 5 Summary and outlook

Overall, the cGAS–STING, TLR, and RLR axes are the three most critical innate immune nucleic acid-sensing pathways in lung cancer, collectively forming a vital network that bridges abnormal nucleic acid recognition, inflammatory cytokine release, and adaptive immunity initiation. On one hand, these pathways can sense abnormal DNA, RNA, and damage-associated molecular patterns derived from tumor cells, inducing the expression of IFN-I and various inflammatory cytokines and chemokines. This promotes DC maturation, enhances antigen cross-presentation, and drives the activation of anti-tumor effector populations such as NK cells, CD8^+^ T cells, and M1-like macrophages, forming a multi-layered immune surveillance system. On the other hand, through the synergistic effects of DNA methylation, genetic mutations, ubiquitin-mediated degradation, metabolic reprogramming, non-coding RNA regulation, and auxiliary inhibitory factors in the immune microenvironment, lung cancer cells can exert continuous negative regulation on these pathways. This gradually transforms them from immune-activating signals into inefficient or even pro-tumorigenic inflammatory outputs, ultimately leading to restricted antigen presentation, insufficient effector lymphocyte infiltration, and the enrichment of immunosuppressive cell populations, thereby establishing a stable state of immune evasion.

Mechanistically, the suppression of innate immunity in lung cancer does not occur in isolation within a single pathway, but is instead manifested as cross-inhibition and synergistic imbalance among multiple nucleic acid sensing axes. The cGAS–STING and RLR pathways can converge at the TBK1–IRF3 node, and TLR and RLR signaling can also interact through inflammatory cytokine networks, the JAK–STAT amplification loop, and metabolic remodeling. Meanwhile, microenvironmental and intracellular negative regulatory factors such as SOCS1/SOCS3, the TRIM family, A20, and CYLD further amplify these inhibitory effects. Therefore, immune evasion in lung cancer is essentially not caused by a single molecular abnormality, but is the comprehensive result of a continuous process: “abnormal nucleic acid sources — pattern recognition receptor imbalance — immune cell functional remodeling — consolidation of the immunosuppressive microenvironment”.

In terms of clinical translation, targeting innate immune pathways has shown considerable potential, but its therapeutic value in lung cancer should be interpreted more cautiously and specifically. Although STING agonists, TLR agonists, and RIG-I agonists may enhance immunotherapy sensitivity by remodeling the “cold tumor” microenvironment, current evidence indicates that their clinical performance remains constrained by multiple translational barriers (241–244). In terms of clinical translation, targeting innate immune pathways has shown considerable potential, but its therapeutic value in lung cancer should be interpreted more cautiously and specifically. Although STING agonists, TLR agonists, and RIG-I agonists may enhance immunotherapy sensitivity by remodeling the “cold tumor” microenvironment, current evidence indicates that their clinical performance remains constrained by multiple translational barriers (57, 245). By comparison, TLR agonists may offer more flexible delivery strategies, such as inhalational administration for DV281 in metastatic NSCLC or intravenous administration for TLR7/8 agonists in PD-(L)1-resistant settings, yet their benefit is still restricted by limited response durability and systemic inflammatory toxicity (246). RIG-I agonists such as CV8102 also demonstrate encouraging immunostimulatory effects, particularly in combination with PD-1 blockade, but RNA instability and inefficient intracellular delivery remain major obstacles to broader clinical application (247). In addition, emerging nanomedicine- or indirect-STING-based strategies, such as TGR-BLDHs-targeted approaches, may help improve delivery efficiency and reduce toxicity, although these approaches remain largely preclinical and still require further evaluation for safety, manufacturability, and translational feasibility in lung cancer (245). At the conceptual level, recent advances have further refined the framework of innate immune regulation in lung cancer. Rather than supporting a purely linear view in which cGAS–STING, TLR, and RLR activation is uniformly antitumor and their suppression uniformly immunosuppressive, newer studies indicate that these pathways function as context-dependent networks whose outputs vary according to cellular compartment, disease stage, signaling strength and duration, and crosstalk with metabolism, inflammation, senescence, and treatment-induced stress (28, 57, 58, 248, 249). In particular, recent work has highlighted the diversity of cellular outcomes downstream of cGAS–STING signaling (28, 57, 58, 248, 249), as well as the compartment-specific divergence of RLR signaling, including the checkpoint-like role of RIG-I in exhausted CD8^+^ T cells and the reactivation of RIG-I/MDA5-MAVS signaling by ADAR1 loss in lung cancer (188). These findings do not merely add new mechanistic details, but collectively shift the current framework from a static pathway-centered model toward a dynamic, compartment-specific, and clinically stratified understanding of innate immune regulation in lung cancer (250). Therefore, the future development of innate immune stimulators in lung cancer should not remain at the level of being merely potential partners of immune checkpoint inhibitors, but should instead be considered within a framework of disease stage, pathological subtype, biomarker stratification, and multimodal combination strategies. Future research should continue to clarify the optimal delivery route, therapeutic window, predictive biomarkers, and resistance-related mechanisms for these agents in different lung cancer contexts (243, 247, 251–253). (**Table 1**).

**Table 1:** Clinical translation of STING, TLR, and RIG-I agonists in lung cancer.

Drug category	Representative agent/system	Route of administration	Main relevance to lung cancer	Clinical stage / key findings	Core limitations
STING agonist	Ulevostinag (MK-1454)	Intratumoral injection	Advanced NSCLC and lung metastases	Phase I/II: In cohorts including NSCLC, disease control was achieved; combination with PD-1 blockade showed preliminary responses	Efficacy is highly dependent on injectable lesions; deep pulmonary lesions are difficult to access directly (254)
STING agonist	ADU-S100 (MIW815)	Intratumoral injection	Advanced treated adenocarcinoma	Phase I: Limited clinical benefit; faced PK bottlenecks	Rapidly degraded by ENPP1 in the lung; poor membrane permeability (254)
STING nanomedicine	TGR-BLDHs	Targeted nanodelivery	Specifically developed for NSCLC	Preclinical: Successfully induced NSCLC cell death and inhibited primary tumor progression	Challenges in scalable manufacturing; pulmonary toxicity still requires evaluation (245)
TLR9 agonist	DV281	Inhalation	Advanced or metastatic NSCLC	Phase Ib: 50% of patients achieved SD; clear evidence of target engagement	No objective response observed in heavily pretreated patients; longer response duration is needed (246)
TLR7/8 agonist	EIK1001	Intravenous injection	Late-stage NSCLC resistant to PD-(L)1 blockade	Phase I/II: Showed preliminary clinical responses in combination with atezolizumab	Systemic inflammatory adverse effects, including fever and chills; narrow tolerability window (255)
RIG-I agonist	CV8102 (RNA)	Intratumoral injection	Advanced solid tumors, including NSCLC	Phase I: Induced systemic immune activation; in combination with PD-1 blockade, showed antitumor activity	Poor RNA stability and low delivery efficiency in the complex lung tumor microenvironment (247)
